# Lipid Disorders in Patients with Renal Failure: Role in Cardiovascular Events and Progression of Chronic Kidney Disease

**DOI:** 10.3390/life16060986

**Published:** 2026-06-11

**Authors:** Maria-Daniela Tanasescu, Andrei-Mihnea Rosu, Alexandru Minca, Maria-Mihaela Grigorie, Delia Timofte, Dorin Ionescu

**Affiliations:** 1 Department of Semiology-Emergency University Hospital, Carol Davila University of Medicine and Pharmacy, 022328 Bucharest, Romania; maria.tanasescu@umfcd.ro (M.-D.T.); dorin.ionescu@umfcd.ro (D.I.); 2 Department of Cardiology, Prof. Dr. Agrippa Ionescu Emergency Hospital, 077015 Balotesti, Romania; 3 Department of Dentistry, Discipline of Endodontics, Faculty of Dentistry, Carol Davila University of Medicine and Pharmacy, 020021 Bucharest, Romania; maria.grigorie@umfcd.ro; 4 Department of Dialysis, Bucharest Emergency University Hospital, 050098 Bucharest, Romania; delia.timofte@gmail.com

**Keywords:** chronic kidney disease, renal failure, dyslipidemia, remnant cholesterol, triglyceride-rich lipoproteins, HDL dysfunction, cardiovascular disease, renal progression, lipid-lowering therapy

## Abstract

Chronic kidney disease (CKD) is associated with a high burden of cardiovascular morbidity and mortality, while lipid disorders in renal failure differ substantially from the LDL-C-centered pattern observed in the general population. This narrative review aimed to synthesize recent evidence on the mechanisms, clinical implications, and therapeutic management of dyslipidemia in patients with renal failure, with emphasis on cardiovascular events and CKD progression. A structured literature search was conducted in PubMed/MEDLINE, Scopus, and Web of Science for publications from January 2018 to April 2026. The review shows that CKD-related dyslipidemia is characterized by triglyceride-rich lipoprotein and remnant particle accumulation, small dense and modified LDL, and dysfunctional HDL within a uremic-inflammatory environment that promotes endothelial injury, vascular calcification, and residual cardiovascular risk. These abnormalities may also contribute to renal lipotoxicity, proteinuria, glomerulosclerosis, tubulointerstitial injury, and fibrosis, although direct causal and therapeutic implications remain incompletely established. Statin-based therapy remains central in non-dialysis CKD, whereas lipid management in dialysis, transplantation, frailty, and severe hypertriglyceridemia requires individualized interpretation. Future risk assessment should integrate lipid, inflammatory, vascular, nutritional, and renal-trajectory markers rather than relying on LDL-C alone.

## 1. Introduction

Chronic kidney disease (CKD) is a major and expanding global health problem, with consequences that extend well beyond the progressive loss of kidney function. Current estimates indicate that approximately 850 million people worldwide live with some form of kidney disease, a figure roughly double the global prevalence of diabetes and higher than that of several other major chronic diseases [1,2]. Despite this scale, CKD remains underdiagnosed in many settings, particularly in low- and middle-income countries, where limited access to screening, early nephrology care, and preventive treatment contributes to delayed recognition and poorer outcomes [1]. Its burden is expected to increase further as populations age and as diabetes, hypertension, obesity, and cardiovascular disease (CVD) become more prevalent.

Recent epidemiological data have shown that CKD prevalence and mortality increased between 1990 and 2021, while the disability-adjusted life-year (DALY) burden also rose over time [3]. Projections indicate that the age-standardized prevalence rate may increase from 8544.81 per 100,000 population in 2022 to 8773.85 per 100,000 population by 2032, while the age-standardized mortality rate may rise from 19.55 to 21.26 per 100,000 population over the same period [3]. CKD has also been linked to substantial premature mortality, with kidney disease projected to become the fifth leading cause of years of life lost globally by 2040 [1,3].

This epidemiological burden is closely connected to cardiovascular risk. CKD is classified according to cause, glomerular filtration rate (GFR), and albuminuria, because declining kidney function and increasing albuminuria identify patients at higher risk for kidney failure, cardiovascular complications, and death [2]. Within this risk profile, dyslipidemia requires specific interpretation. Lipid abnormalities in CKD are not limited to elevated low-density lipoprotein cholesterol (LDL-C), as typically emphasized in the general population. Uremic conditions modify both the concentration and function of circulating lipoproteins, producing a pattern often characterized by increased triglycerides (TG), reduced high-density lipoprotein cholesterol (HDL-C), small dense low-density lipoprotein (sdLDL) particles, and qualitative changes in lipoprotein composition [4].

Cardiovascular risk in renal failure cannot be explained by conventional risk factors alone. As kidney function declines, patients are exposed to hypertension, diabetes, obesity, smoking, and dyslipidemia, together with CKD-specific abnormalities such as albuminuria, inflammation, oxidative stress, vascular calcification, anemia, hypervolemia, left ventricular hypertrophy (LVH), and accumulation of uremic metabolites. In advanced CKD, CVD accounts for approximately half of all deaths, and patients with moderate-to-severe CKD may be more likely to die from cardiovascular causes than to progress to kidney failure requiring kidney replacement therapy (KRT) [5].

CKD-related dyslipidemia has a stage-dependent biological profile. From the early stages of CKD, triglyceride-rich lipoproteins (TRLs) and remnant particles may accumulate because hepatic production increases while peripheral clearance declines. Reduced lipoprotein lipase (LPL) activity, increased apolipoprotein C-III (ApoC-III), impaired hepatic lipase (HL) activity, insulin resistance, and uremic toxins favor the persistence of very-low-density lipoproteins (VLDLs) and intermediate-density lipoproteins (IDLs) in the circulation. LDL-C may remain relatively unchanged, but LDL particles become more atherogenic through a shift toward sdLDL and impaired clearance [6]. High-density lipoprotein (HDL) is also altered in CKD and end-stage kidney disease (ESKD), with impaired reverse cholesterol transport, reduced antioxidant activity, and changes in particle composition, meaning that HDL-C concentration may not accurately reflect HDL function [7].

In a representative general-population analysis, impaired renal function or albuminuria was identified in 10.98% of participants, and higher remnant cholesterol (RC) showed an inverse dose–response association with estimated glomerular filtration rate (eGFR) [8]. These abnormalities fit within a broader molecular setting in which CKD modifies lipoprotein concentration, composition, and function through uremic solute accumulation, post-translational modification, inflammation, and oxidative stress [9]. Lipid disorders in renal failure therefore need to be interpreted as qualitative, functional, and stage-dependent disturbances, rather than as simple deviations in LDL-C alone.

Compared with existing guidelines and previous reviews, the novel contribution of this article is its integrated, stage-specific interpretation of dyslipidemia in renal failure across non-dialysis CKD, dialysis, and kidney transplantation. Current guidelines provide clear therapeutic recommendations, particularly for statin-based cardiovascular prevention, but they necessarily offer less detailed discussion of how uremia, inflammation, remnant lipoproteins, HDL dysfunction, lipid paradox, frailty, dialysis status, and transplant-related metabolic changes interact in clinical interpretation. This review therefore aims to bridge mechanistic evidence, observational risk markers, trial-based treatment data, and practical decision-making. Particular attention is given to areas where evidence remains incomplete, including the clinical use of non-HDL-C, apolipoprotein B, remnant cholesterol, and lipid-related markers of renal progression, so that these emerging concepts are presented as complementary tools rather than replacements for guideline-based care.

Accordingly, this narrative review synthesizes recent evidence on lipid disorders in patients with renal failure, with emphasis on their contribution to cardiovascular events and CKD progression. The review addresses quantitative and qualitative lipid abnormalities, including TRLs, remnant particles, HDL dysfunction, LDL particle modification, inflammation, oxidative stress, and impaired reverse cholesterol transport. It also places these mechanisms within a clinically relevant framework, considering how CKD stage, dialysis status, transplantation, frailty, and the altered cardiovascular risk profile of renal failure may influence lipid interpretation and therapeutic decision-making.

## 2. Narrative Review Design and Literature Search Strategy

This paper was developed as a narrative review examining lipid disorders in patients with renal failure, with particular attention to cardiovascular events and CKD progression. The purpose was to provide a clinically oriented and mechanistically integrated synthesis of a heterogeneous field, rather than a quantitative evidence synthesis. The review therefore connects altered lipid metabolism, uremia-related lipoprotein dysfunction, vascular injury, renal injury pathways, and lipid-lowering strategies within a single interpretive framework.

A structured literature search was conducted in PubMed/MEDLINE, Scopus, and Web of Science. The primary search window covered publications from January 2018 to April 2026. This interval was chosen to capture contemporary evidence on CKD-related dyslipidemia, remnant cholesterol, HDL dysfunction, dialysis- and transplantation-specific lipid patterns, emerging non-statin therapies, and recent guideline updates. Earlier pivotal randomized trials, major guideline documents, and foundational mechanistic studies were also included when they remained necessary for current clinical interpretation, The search strategy combined controlled vocabulary and free-text terms related to kidney disease, lipid abnormalities, cardiovascular outcomes, renal progression, and lipid-lowering therapy. Representative terms included “chronic kidney disease”, “renal failure”, “end-stage kidney disease”, “hemodialysis”, “peritoneal dialysis”, “kidney transplantation”, “dyslipidemia”, “lipoproteins”, “LDL cholesterol”, “HDL dysfunction”, “triglycerides”, “remnant cholesterol”, “apolipoprotein B”, “lipoprotein(a)”, “oxidized LDL”, “cardiovascular events”, “atherosclerosis”, “vascular calcification”, “CKD progression”, “proteinuria”, “renal fibrosis”, “statins”, “ezetimibe”, “PCSK9 inhibitors”, “inclisiran”, “bempedoic acid”, “fibrates”, and “omega-3 fatty acids”. Search terms were used alone and in combination, with Boolean combinations adapted to each database. Reference lists of relevant articles were also screened to identify additional publications directly related to the review topic.

Priority was given to randomized controlled trials, major observational cohort studies, meta-analyses, evidence-based clinical practice guidelines, scientific statements, and expert consensus documents relevant to nephrology, cardiology, lipidology, and transplantation medicine. Mechanistic, translational, and biomarker-focused studies were included when they helped explain the biological links between renal failure, lipoprotein dysfunction, vascular injury, and kidney disease progression. Study selection was guided by clinical relevance, methodological quality, recency, and direct relevance to the manuscript’s central questions. When several publications addressed the same topic, preference was given to the most mature, clinically informative, and methodologically robust source.

Evidence was interpreted according to its type and strength. Randomized outcome trials and guideline documents were used as the main basis for therapeutic positioning. Meta-analyses and large cohort studies were used to summarize clinical associations and risk patterns. Mechanistic and translational studies were used to explain biological plausibility, particularly in areas where clinical evidence remains incomplete. Observational associations, biomarker studies, post hoc analyses, and indirect comparative evidence were interpreted cautiously and were not treated as proof of causality.

Explicit selection boundaries were applied. Publications were generally excluded when they were outside the review scope, duplicated the same study population without adding relevant information, represented superseded interim analyses when more complete reports were available, or consisted of case reports, brief opinion pieces, conference abstracts, or low-informative commentaries. Preference was given to peer-reviewed English-language publications. Narrative reviews were used selectively, mainly to contextualize areas in which primary evidence was heterogeneous or mechanistically fragmented.

Because the article was designed as a narrative review rather than a systematic review, no protocol was prospectively registered, no formal risk-of-bias assessment tool was applied across all included studies, and no pooled meta-analytic estimates were generated. This design allowed broader conceptual integration across trials, guidelines, cohort studies, biomarker evidence, and the mechanistic literature, but it also carries a risk of selection bias. This limitation is particularly relevant for dialysis and kidney transplant populations, in which available evidence is frequently observational, heterogeneous, and influenced by dialysis vintage, residual kidney function, inflammation, protein-energy wasting, immunosuppressive regimens, graft function, comorbidity burden, and confounding by indication. The findings should therefore be read as a structured expert synthesis rather than an exhaustive systematic evidence appraisal. For the same reason, study identification was not reported using a PRISMA flow diagram.

## 3. Lipid Metabolism in Renal Failure: Pathophysiological Background

### 3.1. Altered Lipoprotein Metabolism in CKD

Altered lipoprotein metabolism in CKD is driven mainly by impaired handling of TRLs, rather than by a uniform increase in LDL-C. Hypertriglyceridemia (HTG) is among the most frequent lipid abnormalities in CKD, and its prevalence rises as kidney function declines. Triglyceride (TG) concentrations above 150 mg/dL have been reported in up to 60% of patients with CKD stages 3–5, in more than 60% of patients with nephrotic syndrome, and in approximately 45–50% of dialysis recipients [10]. This pattern reflects a metabolic state in which TRL production, remodeling, and clearance become progressively uncoupled.

Under physiological conditions, intestinal chylomicrons and liver-derived VLDL are hydrolyzed by LPL, generating remnant particles that are cleared by hepatic receptors or further remodeled by HL. In CKD, this pathway is disrupted at several points. Reduced LPL gene expression, lower endothelial LPL availability, diminished apolipoprotein C-II (ApoC-II) and apolipoprotein E support, increased ApoC-III, and impaired glycosylphosphatidylinositol-anchored HDL-binding protein 1 activity reduce TRL lipolysis. Insulin resistance and secondary hyperparathyroidism further suppress LPL function, while reduced HL activity delays remnant remodeling and clearance [11].

The consequence is prolonged circulation of VLDL, IDL, chylomicron remnants, and other apolipoprotein B-containing particles. In advanced CKD and ESKD, this can produce discordance between LDL-C concentration and atherogenic particle burden: LDL-C may be normal or only modestly reduced, while apolipoprotein B-containing particles and small dense LDL become more prominent [12]. This phenotype is relevant because small dense LDL has greater arterial penetrance and higher susceptibility to oxidative modification.

VLDL occupies an important position in this altered metabolic network. It is the main fasting TRL, carries apolipoprotein B, ApoC-II, ApoC-III, apolipoprotein E, and apolipoprotein A-V, and serves as the precursor of IDL and LDL [13]. When VLDL and remnant clearance are delayed, TRL remnants persist in the circulation and may enter the arterial wall, promote foam-cell formation, increase local inflammation, and contribute to residual cardiovascular risk, particularly in patients with CKD, diabetes, obesity, or metabolic syndrome [14].

### 3.2. HDL Dysfunction and Impaired Reverse Cholesterol Transport

HDL-C concentration does not fully reflect HDL function in CKD. As kidney function declines, HDL particles undergo structural and compositional remodeling that weakens reverse cholesterol transport, antioxidant defense, anti-inflammatory activity, and endothelial protection. Reduced lecithin–cholesterol acyltransferase (LCAT) activity and impaired adenosine triphosphate-binding cassette transporter A1-dependent lipidation disturb HDL maturation, favor immature particles, and alter HDL subclass distribution. HDL also becomes enriched in triglycerides, serum amyloid A, ApoC-II, ApoC-III, and inflammatory proteins, while apolipoprotein A-I, apolipoprotein A-II, apolipoprotein M, phospholipids, paraoxonase-1, and glutathione peroxidase are reduced. These changes impair cholesterol efflux and weaken the antioxidant and anti-inflammatory properties normally associated with HDL [15].

Functional studies support the distinction between HDL quantity and HDL quality in CKD. In biopsy-characterized CKD patients, mean cholesterol efflux capacity was reported at 0.83 ± 0.15, while mean oxygen radical absorbance capacity reached 0.86 ± 0.14. Although HDL-C was associated with cholesterol efflux capacity, it did not fully capture HDL functionality. Cholesterol efflux capacity correlated with eGFR, serum albumin, and antioxidant activity, while antioxidant capacity was positively related to serum albumin and negatively related to the urinary protein–creatinine ratio [16].

HDL subclass distribution may add prognostic information. In CKD stages 2–4, lower cholesterol, apolipoprotein A-I, and apolipoprotein A-II contents in small and extra-small HDL subclasses were associated with higher all-cause mortality, whereas total HDL-C and total HDL apolipoproteins were not independently predictive. Extra-small HDL apolipoprotein A-II showed the strongest signal, with each 1-standard deviation increase associated with a 31% lower mortality risk [17]. Cardiovascular outcome data also indicate that HDL particle size, large HDL particle number, HDL-C, and cholesterol efflux capacity may be associated with incident cardiovascular events in CKD, supporting the clinical relevance of HDL architecture and function beyond HDL-C concentration alone [18].

### 3.3. Oxidative Stress, Inflammation, and Uremic Toxins

Oxidative stress and inflammation create the biochemical setting in which lipid abnormalities become more damaging in CKD. As renal clearance declines, uremic toxins accumulate and promote reactive oxygen species (ROS) generation, endothelial dysfunction, vascular inflammation, and post-translational modification of circulating proteins. More than 140 uremic toxins have been identified, and their accumulation is accompanied by carbamylation, guanidinylation, glycation, and oxidation. These modifications have been linked to inflammatory activation, oxidative injury, vascular damage, prothrombotic changes, and fibrosis. In this environment, low-density lipoprotein (LDL) becomes progressively oxidized and carbamylated, while HDL may accumulate symmetric dimethylarginine and serum amyloid A, shifting toward a dysfunctional and pro-inflammatory phenotype [19].

These lipid changes develop within a wider vascular injury network. CKD adds non-traditional cardiovascular risk factors, including systemic inflammation, oxidative stress, toxic metabolite accumulation, vascular calcification, anemia, hypervolemia, and LVH. Progression of kidney disease intensifies inflammatory signaling, partly through reduced clearance of mediators such as C-reactive protein, interleukin-6, and tumor necrosis factor-α. At the same time, increased ROS generation and impaired antioxidant defenses enhance lipid, protein, and DNA oxidation, contributing to cardiovascular morbidity and mortality [5].

The endothelium is a major target of this uremic-inflammatory environment. Increased permeability, LDL entry into the vessel wall, LDL oxidation, chemokine release, adhesion molecule expression, leukocyte recruitment, and loss of antithrombotic protection form an early sequence of vascular injury. Uremic serum and toxins such as indoxyl sulfate, p-cresyl sulfate, cyanate, advanced glycation end products, phosphate, uric acid, and asymmetric dimethylarginine promote oxidative stress, inflammation, leukocyte adhesion, impaired endothelial repair, cell death, and thrombosis [20]. Macrophage activation further amplifies vascular damage through ROS production, cytokine release, impaired phagocytic clearance, vascular inflammation, calcification, arterial stiffness, and atherosclerosis [21]. Reduced nitric oxide bioavailability links these molecular changes to impaired vasodilation, endothelial activation, vascular remodeling, and loss of microvascular and macrovascular integrity in CKD [22].

**Figure 1:**
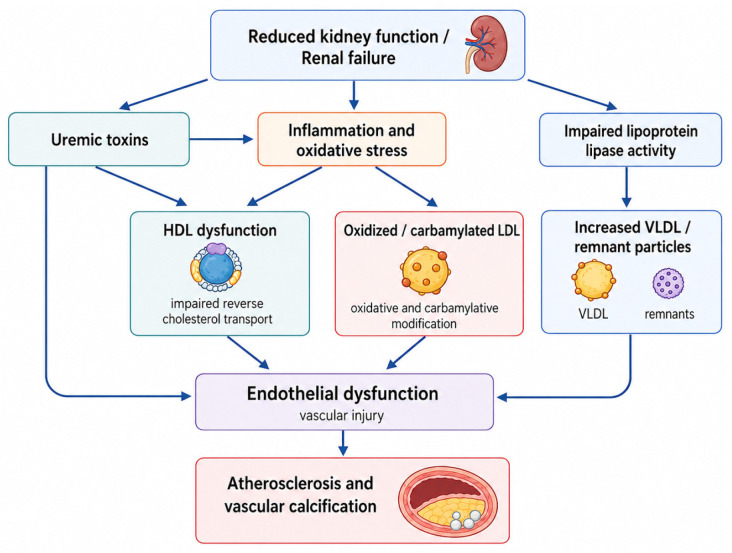
Mechanisms of dyslipidemia in renal failure. Declining kidney function promotes uremic toxin accumulation, inflammation, oxidative stress, impaired triglyceride-rich lipoprotein clearance, HDL dysfunction, and oxidative or carbamylative LDL modification. These abnormalities interact with endothelial dysfunction, vascular inflammation, atherosclerosis, and vascular calcification, contributing to cardiovascular risk in renal failure. Abbreviations: CKD, chronic kidney disease; HDL, high-density lipoprotein; LDL, low-density lipoprotein.

Figure 1 summarizes these interconnected mechanisms, showing how reduced kidney function promotes uremic toxin accumulation, inflammation, oxidative stress, impaired lipoprotein metabolism, HDL dysfunction, and LDL oxidation, with downstream effects on endothelial dysfunction, atherosclerosis, and vascular calcification.

## 4. Patterns of Dyslipidemia Across CKD Stages, Dialysis, and Transplantation

### 4.1. Early and Moderate CKD

Lipid abnormalities may appear before overt renal failure and are often already detectable in early and moderate CKD. In patients with CKD stages 3a–5, lipid parameters vary with renal stage: HDL-C, LDL-C, and albumin decrease as CKD advances, while C-reactive protein increases. Mean HDL-C declined from 45.9 mg/dL in stage 3a to 41.0 mg/dL in stage 5, and LDL-C declined from 125.0 mg/dL to 108.7 mg/dL, indicating that worsening kidney function does not necessarily produce a progressively LDL-C-driven phenotype [23].

In early CKD, the typical lipid pattern is more often characterized by mild HTG, reduced HDL-C, and normal or only slightly increased LDL-C. This reflects early impairment of lipoprotein metabolism, particularly reduced LPL and HL activity, which limits the clearance of TRLs [24,25]. Standard lipid concentrations may also miss qualitative remodeling across the CKD continuum. Lipoprotein composition and activity vary across CKD stages, and these changes may contribute to oxidative stress, inflammation, lipotoxicity, and vascular risk even when LDL-C is not markedly elevated [25].

The triglyceride/HDL-C pattern may have clinical relevance in moderate CKD. In hospitalized CKD patients, median eGFR was 33.6 mL/min/1.73 m^2^, median triglycerides were 114.5 mg/dL, and median HDL-C was 36.5 mg/dL. Higher triglycerides and a higher triglyceride/HDL-C ratio were associated with in-hospital mortality, with proposed thresholds of 115.5 mg/dL for triglycerides and 3.19 for the triglyceride/HDL-C ratio [26]. In diabetic kidney disease, elevated triglycerides and reduced HDL-C are also frequent early abnormalities, whereas LDL-C is often normal or only mildly increased. High triglycerides and low HDL-C have been associated with albuminuria and eGFR decline; each 0.5 mmol/L increase in triglycerides was linked to a 23% higher risk of diabetic kidney disease, while each 0.2 mmol/L increase in HDL-C was linked to a 14% lower risk [27]. Early and moderate CKD therefore already show a stage-sensitive lipid profile, with TRLs, low HDL-C, and particle remodeling appearing before overt renal failure.

### 4.2. Advanced CKD and ESKD

In advanced CKD and ESKD, dyslipidemia is increasingly shaped by uremia, inflammation, altered lipolytic enzyme activity, and nutritional status. Lipid disorders are highly prevalent in hemodialysis (HD) populations; in large dialysis cohorts, dyslipidemia has been reported in up to 82% of patients, with more than half showing HTG, elevated very-low-density lipoprotein cholesterol (VLDL-C), and reduced HDL-C [28]. This pattern reflects impaired lipid handling rather than simple cholesterol excess. Reduced hepatic triglyceride lipase and peripheral LPL activity delay the breakdown of TRLs, while insulin resistance, secondary hyperparathyroidism, uremia-related oxidative stress, and chronic inflammation further promote triglyceride accumulation and HDL dysfunction [28].

HTG remains a prominent abnormality as kidney function deteriorates. Triglyceride concentrations above 150 mg/dL may be present in up to 60% of patients with CKD stages 3–5 and in approximately 45–50% of dialysis recipients [10]. TRL remnants are metabolically active and may contribute to lipid accumulation, oxidative stress, inflammation, endothelial dysfunction, and renal fibrosis, thereby linking advanced CKD with residual cardiovascular and renal risk [10].

Inflammation modifies lipid interpretation in late-stage CKD. Chronic low-grade inflammation worsens with renal failure and is driven by reduced glomerular filtration, immune activation, oxidative stress, intestinal dysbiosis, uremic toxin retention, dialysis-related factors, and protein-energy wasting (PEW). C-reactive protein is elevated in more than half of patients from CKD stage 3 onward, with a higher incidence in ESKD. Oxidative stress further contributes to dyslipidemia by altering lipid and protein components, reducing clearance of TRLs, inhibiting LPL function, and lowering HDL concentrations [29].

This inflammatory and malnutrition-prone background also complicates the prognostic meaning of lipid values in dialysis. In advanced CKD and ESKD, lower lipid concentrations may reflect inflammation, illness severity, PEW, or dialysis-related metabolic stress rather than lower vascular risk [30,31]. Therefore, lipid values in advanced CKD and ESKD should be interpreted alongside inflammation, nutritional status, dialysis exposure, and residual cardiovascular risk.

### 4.3. Hemodialysis and Peritoneal Dialysis

Lipid abnormalities are frequent in both HD and peritoneal dialysis (PD), but their pattern differs by modality. In HD, dyslipidemia is usually characterized by HTG, elevated VLDL-C, reduced HDL-C, and impaired HDL function. These changes are linked to reduced LPL and LCAT activity, delayed clearance of triglyceride-rich particles, uremia-related oxidative stress, chronic inflammation, and the metabolic effects of ESKD [28].

PD patients often show a more atherogenic lipid profile than HD patients, particularly with higher total cholesterol, triglycerides, and LDL-C. Direct modality comparisons indicate that these lipid fractions tend to be higher after PD than after HD, although HDL-C may be lower in HD populations. This pattern indicates that dialysis modality influences lipid metabolism through different mechanisms rather than producing a uniform dialysis-related phenotype [32].

The more atherogenic profile observed in PD is partly explained by continuous glucose absorption from dialysis solutions. This glucose load may contribute to hyperglycemia, insulin resistance, hepatic lipogenesis, and increased production of VLDL and LDL particles. Peritoneal protein loss may further stimulate hepatic lipoprotein synthesis and alter HDL metabolism. However, the relationship between glucose exposure and lipid changes is not linear across all lipid parameters. Higher glucose absorption has been associated most consistently with lower HDL-C, while associations with total cholesterol, LDL-C, triglycerides, and lipoprotein(a) (Lp(a)) appear less uniform [33,34].

Beyond cardiovascular risk, dyslipidemia in PD may also relate to modality-specific outcomes. Atherogenic lipid patterns have been associated with all-cause mortality, cardiovascular mortality, and peritonitis risk, indicating that lipid disturbances in PD may reflect both systemic metabolic risk and local peritoneal vulnerability [35]. Lipid interpretation in dialysis should therefore account for modality, residual kidney function, glucose exposure, protein loss, inflammation, and nutritional status, rather than applying the same assumptions to all patients receiving KRT.

### 4.4. Kidney Transplant Recipients

Post-transplant dyslipidemia represents a distinct pattern within the CKD continuum. Kidney transplantation improves uremia, but it also introduces metabolic and pharmacological drivers of lipid disturbance. Dyslipidemia has been reported in approximately three-quarters of kidney transplant recipients, often with the highest incidence during the first post-transplant year and persistence as a long-term cardiovascular risk factor. In recipients treated with tacrolimus-based immunosuppression, transplantation was associated with increases in total cholesterol and LDL-C, while HDL-C improved and triglycerides tended to decrease. Statin therapy appeared to attenuate the rise in total cholesterol and LDL-C and further improved HDL-C and triglyceride levels, despite post-transplant weight gain and higher glycated hemoglobin values [36].

The mechanisms are multifactorial. Post-transplant dyslipidemia may develop de novo or persist from pre-existing CKD and dialysis-related lipid abnormalities. The usual phenotype includes increased total cholesterol, LDL-C, VLDL-C, and triglycerides, with variable HDL-C changes. Immunosuppressive therapy is a major contributor. Glucocorticoids increase hepatic lipogenesis, stimulate cholesterol synthesis, inhibit LPL and HL activity, and reduce LDL receptor expression. Calcineurin inhibitors, particularly cyclosporine, impair cholesterol clearance and promote TRL accumulation, whereas mammalian target of rapamycin inhibitors have stronger dyslipidemic effects, increasing LDL-C, VLDL-C, apolipoprotein B-containing particles, and triglycerides [37].

Post-transplant dyslipidemia should also be interpreted within the wider framework of metabolic syndrome. Central obesity, hypertension, impaired glucose metabolism, and dyslipidemia may persist or emerge after transplantation, reflecting pre-existing cardiometabolic risk, sedentary behavior, diet, and immunosuppressive exposure. Metabolic syndrome after transplantation has been associated with a two- to threefold higher risk of composite vascular outcomes, including cardiovascular events and graft loss, during long-term follow-up [38]. Weight gain adds to this risk profile. Adipose tissue commonly increases after transplantation, partly because of improved general health, increased appetite, and the metabolic effects of glucocorticoids and calcineurin inhibitors. Average post-transplant weight gain may range from 10% to 35% of baseline body weight, with the greatest increase during the first year; visceral adiposity may promote insulin resistance, inflammation, endothelial dysfunction, and chronic graft injury [39].

Residual renal dysfunction further modifies lipid-related risk. Transplant recipients retain CKD-associated cardiovascular risk factors, including proteinuria, suboptimal graft function, inflammation, anemia, and prior dialysis exposure. In this setting, lipid abnormalities may contribute to atherosclerotic risk and graft injury through podocyte damage, proteinuria, platelet activation, and inflammation. Lipid assessment after kidney transplantation should therefore be integrated with immunosuppressive regimen, weight trajectory, metabolic syndrome status, graft function, proteinuria, and global cardiovascular risk [40].

The main lipid abnormalities described across the CKD continuum are summarized in Table 1.

## 5. Dyslipidemia and Cardiovascular Events in Renal Failure

### 5.1. Atherosclerotic Cardiovascular Disease in CKD

Atherosclerotic cardiovascular disease (ASCVD) is highly prevalent across the CKD continuum and includes coronary artery disease (CAD), acute coronary syndrome, ischemic and hemorrhagic stroke, and peripheral artery disease (PAD). CKD increases cardiovascular risk even after adjustment for traditional factors such as hypertension, diabetes, smoking, and dyslipidemia. As kidney function declines, the probability of CAD rises, and patients with CKD stages G3a–G4 have a two- to threefold higher cardiovascular mortality risk than individuals without comparable renal impairment [41]. Ischemic heart disease is frequent before dialysis initiation, while advanced coronary atherosclerotic lesions and coronary artery calcification become more common with CKD progression [41].

The vascular burden extends beyond the coronary bed. CKD is associated with higher rates of stroke and PAD, reflecting systemic arterial injury. Stroke risk increases as eGFR declines and albuminuria rises, while PAD forms part of the broader major adverse cardiovascular event (MACE) spectrum observed in CKD populations [42].

Atherogenic lipoproteins contribute to this risk, although standard lipid markers may underestimate their impact. In CKD, LDL-C and total cholesterol may be normal or only modestly elevated, while LDL particles often shift toward a small dense phenotype with greater arterial penetration and susceptibility to oxidation [4]. TRLs and their remnants may further promote endothelial inflammation, ROS generation, cytokine production, and complement activation [4]. Apolipoprotein B (ApoB)-containing particles, including VLDL, IDL, LDL, and Lp(a), may therefore better reflect atherogenic particle burden than LDL-C alone. Elevated ApoB, a higher ApoB/apolipoprotein A-I ratio, and Lp(a) accumulation have been linked to cardiovascular events and CKD progression, supporting their use as complementary markers for risk stratification in renal populations [12].

### 5.2. Non-Traditional Cardiovascular Mechanisms

Cardiovascular events in renal failure are not explained only by classical atherosclerotic risk factors or lipid concentrations. CKD creates a non-traditional cardiovascular environment characterized by uremic toxins, chronic inflammation, oxidative stress, endothelial dysfunction, vascular calcification, procoagulant activation, renin–angiotensin–aldosterone system activation, sympathetic overactivity, and anemia. These mechanisms help explain why heart failure, arrhythmias, acute coronary syndrome, stroke, and PAD remain frequent even when conventional risk factors are addressed [42].

Uremic toxins amplify vascular inflammation through direct effects on macrophage activation and endothelial injury. Indoxyl sulfate has been described as an independent cardiovascular risk factor in CKD and contributes to oxidative stress, inflammatory signaling, endothelial activation, and accelerated atherosclerosis. In this environment, activated macrophages release tumor necrosis factor-α, interleukin-1β, interleukin-6, chemokines, and ROS, sustaining endothelial dysfunction, vascular calcification, arterial stiffening, and atherosclerotic progression [21,42].

Vascular calcification distinguishes CVD in renal failure from ordinary atherosclerosis. It is an active biological process involving calcium–phosphate deposition, osteochondrogenic transformation of vascular smooth muscle cells, loss of calcification inhibitors such as matrix Gla protein and fetuin-A, oxidative stress, inflammation, and mineral metabolism disorders. Intimal calcification, linked to atherosclerotic plaques, and medial calcification, associated with arterial stiffness, both contribute to impaired vascular compliance and higher cardiovascular mortality [43].

These vascular processes interact with myocardial remodeling. Uremic cardiomyopathy is characterized by LVH, diastolic dysfunction, myocardial fibrosis, arterial stiffening, coronary atherosclerosis, and coronary calcification. Uremic toxins, inflammation, oxidative stress, anemia, hypervolemia, insulin resistance, and CKD–mineral and bone disorder contribute to myocardial damage, reduced cardiac efficiency, arrhythmias, heart failure, and sudden cardiac death [44]. Uremic toxin-driven calcification further links indoxyl sulfate, p-cresyl sulfate, advanced glycation end-products, trimethylamine N-oxide, hyperphosphatemia, fibroblast growth factor 23 excess, Klotho deficiency, endothelial dysfunction, and matrix remodeling within overlapping inflammatory–oxidative and mineral–metabolic injury patterns [45].

### 5.3. The Lipid Paradox in Dialysis Patients

In dialysis patients, lipid values do not always carry the same prognostic meaning as in the general population. Although higher cholesterol and LDL-C usually predict atherosclerotic risk outside renal failure, HD cohorts often show an inverse association between lipid levels and mortality. In a large incident HD population, higher time-dependent total cholesterol, HDL-C, non-HDL cholesterol, and LDL-C were associated with lower all-cause, cardiovascular, and non-cardiovascular mortality. This relationship persisted after adjustment for inflammation/malnutrition status, statin use, and geographic variation, indicating that the lipid paradox in HD is unlikely to reflect a single confounder [30]. This finding is clinically relevant because cholesterol in HD should be interpreted as a time-varying marker rather than a fixed baseline exposure. A decline in cholesterol during follow-up may reflect worsening inflammation, protein-energy wasting, intercurrent illness, fluid shifts, or treatment changes, whereas stable or higher cholesterol may partly identify patients with better nutritional reserve. Therefore, the inverse association between cholesterol and mortality should not be read as evidence that cholesterol is protective, but as a sign that measured lipid values in HD are strongly shaped by nutritional and inflammatory status.

Several mechanisms may contribute to this pattern. Low lipid values in HD may reflect dilutional hypolipidemia, repeated fluid shifts, dialysis-related changes in measured lipid fractions, lipid-lowering therapy, or the combined effects of malnutrition, systemic inflammation, and catabolic illness. In this setting, LDL-C becomes a less reliable stand-alone marker of cardiovascular risk, while HTG remains a more typical feature of uremic dyslipidemia [46].

The paradox is also observed in acute cardiovascular settings. Among HD patients hospitalized with acute coronary syndrome, LDL-C and LDL-C below 70 mg/dL did not predict short-term or one-year mortality. Survival correlated positively with serum albumin and total cholesterol and inversely with inflammatory markers such as C-reactive protein and the neutrophil-to-lymphocyte ratio, suggesting that nutritional and inflammatory status may be more informative than LDL-C alone in this setting [47].

PEW helps explain why lower cholesterol may indicate higher risk rather than protection. In maintenance HD, PEW was associated with a higher incidence of MACE and all-cause mortality. Thresholds including total cholesterol below 3.4 mmol/L, albumin below 38 g/L, and prealbumin below 280 mg/L identified patients at increased cardiovascular risk, supporting the interpretation of low cholesterol as a marker of catabolic vulnerability in dialysis populations [48]. For this reason, the lipid paradox should not be used to dismiss ASCVD prevention in dialysis patients. Rather, it supports a more cautious interpretation of lipid values together with albumin, inflammatory markers, nutritional status, dialysis vintage, and clinical trajectory.

For general clinical practice, the lipid paradox should be approached as a warning against isolated lipid interpretation, not as a separate treatment principle. Low cholesterol in HD should prompt assessment of nutritional decline, inflammation, recent illness, dialysis-related changes, and treatment history, while ASCVD prevention should still be guided by cardiovascular history, CKD stage, dialysis status, frailty, life expectancy, and patient preference. Putative circadian or lifestyle-related influences may further affect lipid metabolism in CKD, but their specific contribution to the lipid paradox remains insufficiently defined and should be interpreted cautiously.

### 5.4. Lipoprotein Biomarkers Beyond LDL-C

In renal failure, LDL-C alone may not adequately reflect lipid-related cardiovascular risk. Non-high-density lipoprotein cholesterol (non-HDL-C) and apolipoprotein B (ApoB) are increasingly relevant because they capture atherogenic particle burden more broadly than LDL-C. In CKD, lipid assessment may therefore include LDL-C, non-HDL-C, and ApoB, particularly in advanced stages where baseline cardiovascular risk is high and LDL-C may be discordant with particle burden [42].

ApoB is informative because each ApoB-containing particle has atherogenic potential, including VLDL, IDL, LDL, and Lp(a). In end-stage renal disease, LDL-C may appear normal despite elevated ApoB and predominance of small dense LDL particles. A higher ApoB/apolipoprotein A-I ratio has been proposed as a stronger cardiovascular risk marker than the LDL-C/HDL-C ratio, while Lp(a) accumulation in CKD may reflect impaired renal clearance and adds atherogenic and prothrombotic risk [12].

Triglyceride-rich remnants and RC also add information beyond conventional lipid fractions. RC represents the cholesterol content of TRLs, including very-low-density and IDL in fasting conditions. Higher RC has been associated with greater odds of CKD, lower eGFR, and progression toward end-stage renal disease in diabetic kidney disease, supporting its potential value as a residual risk marker [49].

HDL assessment should also extend beyond HDL-C concentration. During chronic inflammation and oxidative stress, HDL particles may lose cholesterol efflux capacity, antioxidant activity, anti-inflammatory function, and endothelial protection. Dysfunctional HDL may even promote inflammation, oxidative stress, endothelial damage, and foam-cell formation despite apparently acceptable HDL-C values [50]. Oxidized lipid cargo adds another dimension: Lp(a) carries oxidized phospholipids, and elevated Lp(a) and oxidized phospholipid measures have been associated with extracoronary vascular disease and major adverse limb events, supporting the relevance of oxidized lipoprotein biology in systemic vascular risk [51].

Because several of these markers are not routinely reported in all laboratories, their use in CKD should follow a pragmatic stepwise approach. Table 2 summarizes a practical approach for daily use.

The principal lipid-related and lipid-interacting mechanisms through which renal failure may contribute to cardiovascular events are summarized in Table 3.

## 6. Lipid Disorders and Progression of CKD

### 6.1. Lipotoxicity and Renal Cellular Injury

Lipotoxicity provides a biologically plausible mechanistic link between dyslipidemia and renal cellular injury, although its direct contribution to CKD progression in humans remains difficult to isolate from diabetes, obesity, inflammation, proteinuria, and other metabolic or hemodynamic factors. In CKD, lipid accumulation may affect both glomerular and tubular compartments through increased lipid uptake, enhanced local synthesis, impaired oxidation, and reduced efflux. Cholesterol, free fatty acids, triglycerides, sphingolipids, and ceramides can accumulate in cytosolic lipid droplets, mitochondria, lysosomes, and the endoplasmic reticulum, where they disturb organelle function and activate oxidative, inflammatory, apoptotic, complement-related, and profibrotic pathways. This process is particularly relevant in proximal tubular epithelial cells, which depend heavily on mitochondrial fatty acid oxidation (FAO) for adenosine triphosphate production. When FAO declines, lipid overload may contribute to mitochondrial dysfunction, reduced energy availability, oxidative stress, and cell injury [52,53].

Renal lipid uptake is mediated partly by CD36, fatty acid transport proteins, fatty acid-binding proteins, and lipoprotein receptors expressed in tubular cells, podocytes, and mesangial cells. In lipid-rich states, oxidized LDL and excess free fatty acids can activate inflammatory pathways, increase ROS generation, depolarize mitochondrial membranes, deplete ATP, and trigger apoptosis [53]. Lipotoxicity also interacts with obesity-related inflammation, where adipose-derived cytokines, leptin, interleukin-6, tumor necrosis factor-α, monocyte chemoattractant protein-1, and oxidative signaling promote podocyte dysfunction, tubular stress, and progressive renal structural injury [54].

### 6.2. Dyslipidemia, Proteinuria, and Glomerulosclerosis

Dyslipidemia may aggravate proteinuric kidney disease by affecting the glomerular filtration barrier. In diabetic kidney disease, high triglycerides, reduced HDL-C, increased small dense LDL-C, and triglyceride-rich lipid deposition have been associated with albuminuria, eGFR decline, and worsening glomerular injury. Lipid accumulation within podocytes disrupts cellular homeostasis, impairs mitochondrial energy metabolism, increases ROS generation, and alters actin cytoskeletal organization. These changes may favor foot process effacement, podocyte detachment, and increased glomerular permeability [27].

Glomerular lipid deposition may also involve mesangial and capillary structures. In disorders marked by abnormal lipoproteins or impaired lipid handling, lipid deposits have been described together with mesangial expansion, basement membrane changes, podocyte injury, proteinuria, and progressive glomerulosclerosis [52]. Obesity-related glomerulopathy provides a related structural model, in which glomerular hypertrophy, podocytopathy, mesangial matrix expansion, and focal segmental glomerulosclerosis develop alongside metabolic stress and lipotoxic injury [54]. At the cellular level, podocyte lipid overload and oxidative stress may amplify one another, promoting mitochondrial injury, inflammatory signaling, extracellular matrix accumulation, and loss of filtration barrier integrity [55].

### 6.3. Tubulointerstitial Injury and Fibrosis

Tubulointerstitial fibrosis is a common pathway of CKD progression, and disturbed lipid handling in tubular epithelial cells appears to contribute to this process. Injured tubular cells have high energetic demands and rely strongly on mitochondrial FAO. When FAO declines, lipid droplets may accumulate and promote mitochondrial dysfunction, endoplasmic reticulum stress, ROS generation, and fibrogenic activation. In experimental CKD models and human CKD samples, increased 2-arachidonoylglycerol was associated with tubular lipid deposition, reduced ATP production, lower FAO-related protein expression, and increased extracellular matrix markers. Preservation or supplementation of monoacylglycerol lipase (MAGL) improved FAO, limited tubular lipid-mediated toxicity, and attenuated renal fibrosis in experimental settings [56].

Defective lipid clearance within tubular epithelial cells may further promote fibrotic remodeling. Lipophagy normally degrades lipid droplets and supports fatty acid mobilization for mitochondrial β-oxidation. In CKD-related tubulointerstitial fibrosis, increased leucyl-tRNA synthetase 1 (LARS1) was associated with more severe interstitial fibrosis, while LARS1 deficiency reduced lipid deposition and fibrosis. Transforming growth factor-β1 activated LARS1-mediated mTORC1 signaling, suppressed lipophagy, increased tubular lipid accumulation, and promoted epithelial–mesenchymal transition in mechanistic models [57].

Lipid metabolism also intersects with immune activation. In diabetic kidney disease, impaired macrophage cholesterol efflux may favor macrophage lipid accumulation and foam-cell transformation. These macrophages release tumor necrosis factor-α, interleukin-1β, and monocyte chemoattractant protein-1, potentially sustaining inflammation, macrophage recruitment, tubular atrophy, and interstitial fibrosis [58]. Transcriptomic analyses of kidney fibrosis samples have also identified lipid metabolism-related gene signatures linked with immune-cell infiltration, inflammatory activation, and distinct fibrosis phenotypes, supporting an interaction between lipid metabolic remodeling and tubulointerstitial immune–fibrotic injury rather than proving a direct causal pathway in clinical CKD progression [59].

### 6.4. Clinical Evidence Linking Lipids to CKD Progression

Clinical evidence links lipid abnormalities with CKD progression, although causality remains difficult to establish because much of the available evidence comes from observational cohorts, cross-sectional analyses, or post hoc trial datasets. These data should therefore be interpreted as evidence of association and risk stratification, rather than proof that remnant cholesterol, triglycerides, or composite lipid indices directly cause CKD progression. Residual confounding is likely, particularly from diabetes control, obesity, insulin resistance, proteinuria, inflammation, nutritional status, baseline eGFR, medication use, and cardiovascular comorbidity. Reverse causality is also possible, because declining kidney function itself alters triglyceride-rich lipoprotein clearance and may increase remnant lipoprotein burden. In patients with type 2 diabetes mellitus (T2DM)-related CKD, RC was independently associated with renal function progression. RC values above 0.56 mmol/L were linked to a higher risk of a renal composite endpoint and rapid renal function decline, whereas triglycerides were associated only with the renal composite endpoint, and total cholesterol showed risk mainly in patients with proteinuria ≥ 0.5 g/day [60]. In biopsy-confirmed diabetic nephropathy, higher RC was also associated with worse renal survival and progression to ESKD. Patients in the highest RC quartile had a 2.857-fold higher adjusted risk of ESKD than those in the lowest quartile, and each one-standard-deviation increase in RC was associated with higher ESKD risk [61].

Broader population cohorts also indicate that lipid-derived indices may add prognostic information. In a four-year longitudinal analysis of adults without CKD at baseline, triglycerides, RC, and the atherogenic index of plasma (AIP) were positively associated with rapid renal function decline, whereas LDL-C and HDL-C showed inverse associations. AIP had the strongest association with both incident CKD and rapid decline [62]. In a post hoc analysis of the ACCORD trial, higher RC was associated with albuminuria and worsening renal function over seven years, but not with renal failure after multivariable adjustment. This pattern indicates that RC may be more closely related to earlier adverse renal outcomes than to hard kidney failure endpoints [63]. Such findings are hypothesis-generating and may identify patients with higher cardiometabolic and renal risk, but they do not establish that lowering RC will slow eGFR decline or prevent kidney failure.

Composite lipid indices have produced similar signals in cardiometabolic populations. In patients with cardiovascular–kidney–metabolic syndrome, the non-HDL-to-HDL cholesterol ratio, natural logarithm of RC, and cholesterol–HDL–glucose index were associated with rapid kidney function decline, with lnRC showing the best discriminative performance [64]. A recent multicenter Chinese T2DM cohort also found that each 0.1 mmol/L RC increase was associated with 43% higher adjusted odds of diabetic kidney disease, although the cross-sectional design limits inference on progression [65]. Thus, these findings support RC, TRL burden, and composite lipid indices as clinically relevant markers of renal risk. At present, however, they should not be interpreted as validated therapeutic targets for renoprotection. No lipid-lowering trial has yet demonstrated that selective lowering of RC or triglyceride-rich lipoprotein burden improves renal outcomes as a primary endpoint. However, interventional studies are still needed to determine whether direct reduction in these abnormalities can modify albuminuria, eGFR decline, renal fibrosis, or progression to kidney failure.

**Figure 2:**
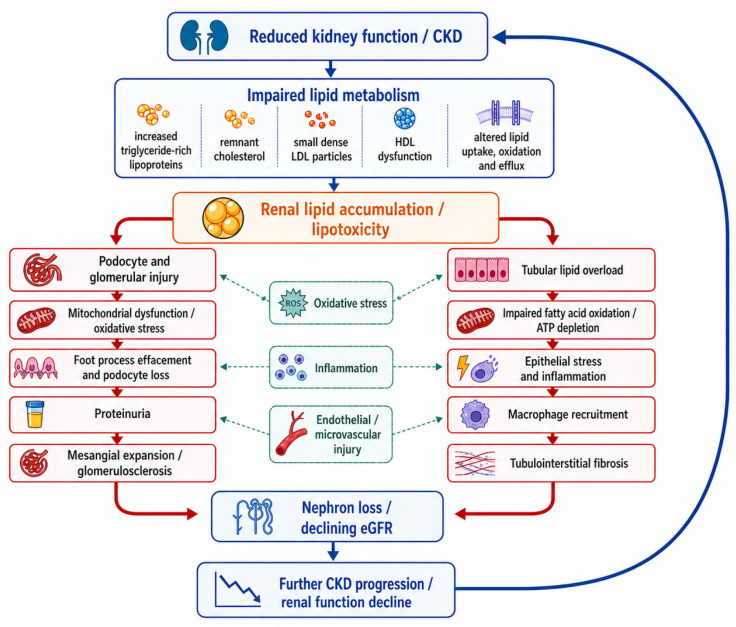
Bidirectional relationship between dyslipidemia and CKD progression. Reduced kidney function promotes impaired lipid metabolism, including triglyceride-rich lipoprotein accumulation, remnant cholesterol, small dense LDL particles, HDL dysfunction, and altered lipid uptake, oxidation, and efflux. These abnormalities may contribute to renal lipid accumulation and lipotoxicity, with downstream effects on podocytes, glomerular structures, tubular epithelial cells, mitochondrial function, inflammation, oxidative stress, microvascular injury, tubulointerstitial fibrosis, nephron loss, and declining eGFR. Progressive CKD may then further aggravate lipid metabolic disturbances, creating a self-reinforcing injury cycle. Abbreviations: ATP, adenosine triphosphate; CKD, chronic kidney disease; eGFR, estimated glomerular filtration rate; HDL, high-density lipoprotein; LDL, low-density lipoprotein; ROS, reactive oxygen species.

Figure 2 summarizes the proposed bidirectional relationship between dyslipidemia and CKD progression. Impaired lipid metabolism may contribute to renal lipid accumulation, glomerular injury, tubular lipid overload, inflammation, oxidative stress, endothelial and microvascular injury, nephron loss, and declining eGFR, while progressive loss of kidney function further worsens lipid homeostasis.

## 7. Therapeutic Management of Dyslipidemia in Renal Failure

### 7.1. Statins and Statin–Ezetimibe Therapy

Statins remain the best-supported lipid-lowering therapy for cardiovascular risk reduction in patients with CKD, although treatment decisions should account for CKD stage and dialysis status. In non-dialysis CKD, especially stages G3–G5, statin therapy or statin–ezetimibe therapy is supported by the high baseline risk of ASCVD. The strongest randomized evidence for this approach shows that combined LDL-C lowering with simvastatin plus ezetimibe reduces major atherosclerotic events in patients with CKD, including a large non-dialysis subgroup. However, this evidence should be interpreted with important limitations: the absolute risk reduction was modest, benefit was not statistically significant among patients already receiving dialysis at baseline, and prespecified renal progression outcomes were not significantly reduced. Thus, statin–ezetimibe therapy should be presented primarily as an ASCVD-prevention strategy, not as a proven renoprotective intervention [66]. Current recommendations therefore favor risk-based treatment rather than decisions based only on LDL-C, since LDL-C becomes less reliable as kidney function declines and may be influenced by inflammation, malnutrition, dialysis status, and altered lipoprotein composition [6].

The benefit is less certain once chronic dialysis has started. Randomized trials in HD populations did not show clear cardiovascular benefit when statins were initiated after dialysis onset [67,68]. This explains why most guidelines do not recommend routine de novo initiation in dialysis-dependent CKD. In this population, the weaker treatment signal probably reflects a different cardiovascular phenotype, with a greater contribution of sudden cardiac death, heart failure, vascular calcification, inflammation, malnutrition, and competing non-atherosclerotic risks. Continuation may still be reasonable in patients already receiving statins before dialysis, particularly when life expectancy, transplant candidacy, and atherosclerotic risk support ongoing prevention. Recent real-world data from a large HD cohort associated statin use after dialysis initiation with lower risk of stroke, myocardial infarction, and all-cause mortality, with stronger benefit among prior statin users, although residual confounding cannot be excluded [6,69,70].

ESC/EAS dyslipidaemia guidance also supports a risk-based interpretation of lipid management in CKD. Moderate CKD is classified as a high cardiovascular risk condition, whereas severe CKD is classified as very-high risk, reinforcing the need to interpret lipid treatment within the patient’s global ASCVD-risk profile rather than by LDL-C concentration alone. For moderate-to-severe non-dialysis CKD, statin or statin–ezetimibe therapy is recommended for ASCVD risk reduction, while continuation of pre-existing statin-based therapy may be considered when dialysis is initiated, particularly in patients with established ASCVD. In contrast, de novo statin initiation is not recommended in dialysis-dependent CKD patients without ASCVD, reflecting the weaker randomized evidence in this population. Recent ESC/EAS updates preserve this risk-based framework while incorporating newer evidence on LDL-C-lowering strategies, risk estimation, Lp(a), hypertriglyceridemia, and non-statin therapies [71,72].

In diabetic kidney disease, lipid management should be integrated with broader cardiovascular and kidney protection, including blood pressure control, glycemic management, weight management, smoking cessation, and kidney-protective therapies. Atorvastatin is commonly proposed as first-line therapy, while dose titration and statin choice require caution at low eGFR and in patients receiving interacting drugs [70]. These measures should be viewed as part of global cardiometabolic risk reduction; they should not be presented as evidence that statin-based lipid lowering directly prevents CKD progression.

### 7.2. PCSK9 Inhibitors and Emerging LDL-Lowering Therapies

Proprotein convertase subtilisin/kexin type 9 (PCSK9) inhibitors provide an additional LDL-C-lowering option for CKD patients who remain above lipid targets despite statin therapy, with or without ezetimibe, or who have very high ASCVD risk. In a CKD cohort treated with evolocumab or alirocumab, LDL-C, total cholesterol, and Lp(a) decreased significantly after three months across CKD stages, including stages 4–5. Serum creatinine and eGFR remained stable, and adverse drug reactions did not differ significantly by kidney function group, supporting short-term safety in selected high-risk CKD patients [73].

Alirocumab, evolocumab, and inclisiran reduce LDL-C effectively in mild-to-moderate CKD, but evidence remains limited in advanced CKD, ESKD, dialysis, and kidney transplant populations. Circulating PCSK9 levels are also difficult to interpret in CKD because they may be influenced by glycemic status, proteinuria, renal function, dialysis modality, lipid-lowering treatment, and sampling conditions. For this reason, PCSK9 concentration should not be treated as a simple cardiovascular risk marker in renal populations [74].

Inclisiran may be useful when statins are insufficient or poorly tolerated. In a small real-world CKD series, twice-yearly inclisiran produced nearly 50% reductions in total cholesterol and LDL-C over 12 months, without significant eGFR change or drug-related adverse effects [75]. Bempedoic acid offers another oral option for statin-intolerant high-risk patients, although renal safety requires careful interpretation. Small early increases in creatinine and blood urea nitrogen have been reported, but available trial analyses described these as laboratory changes rather than clinically meaningful kidney injury [76]. Nevertheless, these emerging LDL-lowering therapies should currently be positioned as adjuncts for ASCVD risk management in selected CKD patients, not as therapies with proven disease-modifying effects on CKD progression.

### 7.3. Fibrates and Triglyceride-Lowering Strategies

HTG is frequent in CKD and reflects impaired clearance of TRLs, reduced LPL activity, increased inhibitory apolipoproteins, insulin resistance, inflammation, and uremia-related metabolic disturbance. Persistent triglyceride elevation may contribute to residual cardiovascular risk and may interact with renal lipotoxicity, inflammation, and fibrosis [10]. However, the clinical evidence remains mainly associative, and it is not yet established that lowering triglycerides or TRL burden slows albuminuria progression, eGFR decline, renal fibrosis, or progression to kidney failure. Triglyceride-lowering therapy in CKD therefore requires caution. Conventional fibrates, particularly fenofibrate, may increase serum creatinine and reduce eGFR, while renal clearance limits their use in advanced CKD. Myopathy risk also increases when fibrates are combined with statins or used in patients with reduced renal function [10].

Pemafibrate, a selective peroxisome proliferator-activated receptor-α modulator, has been proposed as a potentially better tolerated alternative because it is mainly hepatically metabolized rather than principally renally excreted [10,77]. In long-term post-marketing surveillance, pemafibrate reduced triglycerides by approximately 35%, with a low frequency of renal-related adverse drug reactions and no reported rhabdomyolysis events [77]. In patients with type 2 diabetes and CKD, switching from conventional fibrates to pemafibrate was associated with improved eGFR, particularly after fenofibrate discontinuation, although this effect was less evident in severe renal dysfunction or macroalbuminuria [78].

Severe HTG should be managed separately from routine cardiovascular prevention because pancreatitis risk changes the therapeutic priority. Acute pancreatitis risk rises steeply when triglycerides exceed approximately 10 mmol/L, or 885 mg/dL, and becomes particularly high in chylomicronemia, uncontrolled diabetes, alcohol excess, pregnancy, or medication-induced HTG [79]. In these patients, treatment should prioritize rapid triglyceride reduction, correction of secondary causes, and recurrence prevention [79].

### 7.4. Omega-3 Fatty Acids and Residual Lipid Risk

Omega-3 fatty acids may be considered in renal failure mainly for residual TRL risk, but clinical interpretation should distinguish purified eicosapentaenoic acid from mixed fish-oil preparations. In REDUCE-IT RENAL, icosapent ethyl at 4 g/day reduced fatal and nonfatal ischemic events across baseline kidney-function categories in statin-treated patients with elevated triglycerides. The greatest absolute benefit was observed in patients with eGFR < 60 mL/min/1.73 m^2^, in whom the primary composite endpoint occurred in 21.8% of treated patients versus 28.9% of placebo-treated patients, with no meaningful change in median eGFR during follow-up. Bleeding-related events were numerically more frequent in the lower eGFR subgroup, so treatment should be individualized in patients with high bleeding risk [80].

The anti-inflammatory rationale is supported by HD data, although findings are not uniform. Fish-oil supplementation reduced C-reactive protein, particularly when baseline C-reactive protein was ≥5 mg/L, but did not significantly reduce interleukin-6 or tumor necrosis factor-α [81]. Nutritional data also indicate that omega-3 status may be suboptimal in CKD. In pre-dialysis and HD patients, higher dietary α-linolenic acid intake was associated with lower systolic blood pressure, C-reactive protein, and carotid intima–media thickness, but the cross-sectional design does not establish therapeutic benefit [82]. Omega-3-based therapy may therefore be useful in selected CKD phenotypes with elevated triglycerides or inflammatory residual risk, but CKD-specific outcome evidence remains formulation-dependent and heterogeneous.

Clinical caution: evidence gaps for non-statin therapies in CKD. Most non-statin lipid-lowering therapies have not been tested in dedicated CKD-specific cardiovascular or renal outcome trials, particularly in advanced CKD, dialysis-dependent CKD, and kidney transplant recipients. PCSK9 inhibitors, inclisiran, bempedoic acid, fibrates, pemafibrate, omega-3 formulations, and emerging ApoC-III- or ANGPTL3-targeted therapies may improve selected lipid parameters or be useful in specific high-risk phenotypes, but their effects on hard cardiovascular outcomes and CKD progression remain incompletely defined in renal populations. At present, these agents should generally be interpreted as adjunctive options for LDL-C lowering, residual lipid-risk management, statin intolerance, or severe hypertriglyceridemia, rather than as therapies with established CKD-specific cardiovascular or renoprotective benefit.

The main pharmacological options for lipid management in renal failure are summarized in Table 4, with emphasis on their practical role, renal-specific cautions, and current evidence limitations.

### 7.5. Individualized Lipid Management Across CKD Stages

Beyond drug selection, lipid management in renal failure should be adapted to clinical phenotype, CKD stage, dialysis or transplant status, cardiometabolic risk profile, frailty, and competing risks. Because evidence for direct renoprotection from lipid-lowering therapy remains limited, lipid treatment should be positioned mainly as ASCVD prevention, residual lipid-risk management, or pancreatitis-risk prevention, depending on the clinical phenotype. These phenotype-based considerations are summarized in Table 5.

The certainty of evidence is not uniform across the CKD continuum. In dialysis-dependent and kidney transplant populations, many data come from heterogeneous observational cohorts rather than dedicated randomized outcome trials. Interpretation is therefore limited by dialysis vintage, dialysis modality, residual kidney function, inflammation, protein-energy wasting, comorbidity burden, immunosuppressive regimen, graft function, proteinuria, diabetes, weight gain, and confounding by indication. For these groups, lipid results and treatment decisions should be individualized and should not be extrapolated directly from non-dialysis CKD or general-population lipid trials.

Lipid management in CKD is primarily risk-based rather than LDL-C-target-driven. Baseline lipid assessment is recommended when CKD is identified, but routine repeated testing is not required for most patients unless the result would change management, adherence assessment, or evaluation of secondary dyslipidemia [2]. In non-dialysis CKD, statin-based therapy remains part of broader cardiovascular and kidney-risk reduction, together with blood pressure control, sodium–glucose cotransporter 2 inhibitor therapy where indicated, glycemic management, smoking cessation, weight management, and physical activity [2,70]. In this framework, kidney-risk reduction mainly reflects integrated CKD care rather than evidence that lipid-lowering therapy directly slows CKD progression.

Dialysis and transplantation require a different clinical reading of lipid results. In dialysis-dependent CKD, continuation of pre-existing lipid-lowering therapy may be appropriate in selected patients, whereas routine de novo statin initiation for primary prevention has weaker support. In older or frail patients, treatment intensity should reflect life expectancy, muscle symptoms, fall risk, drug interactions, nutritional status, polypharmacy, and patient preference [69,83]. Kidney transplant recipients remain a high-risk group, but lipid treatment must account for immunosuppressive regimen, graft function, proteinuria, metabolic syndrome, diabetes, and interaction risk. Statins are generally first-line, while ezetimibe, PCSK9-targeting therapies, bempedoic acid, or other non-statin agents may be considered when additional LDL-C reduction is needed or statin tolerance is limited [84].

**Table 5:** Phenotype-based lipid management across the CKD continuum.

CKD Phenotype/Setting	Preferred Management Logic	Practical Considerations	Main Cautions
Non-dialysis CKD [2,6,70]	Risk-based statin therapy	eGFR, albuminuria, ASCVD, diabetes	LDL-C alone is insufficient for risk interpretation
Dialysis-dependent CKD [2,6,70,73]	Continue pre-existing therapy rather than newly initiate in most cases	ASCVD, transplant candidacy, life expectancy	Weak evidence for de novo statin initiation
Kidney transplant recipients [70,84]	First-line statin therapy with interaction-aware prescribing	Immunosuppression, diabetes, obesity, graft function	Calcineurin inhibitor and mTOR inhibitor interactions
Diabetic kidney disease [70,85]	Integrated cardiometabolic management	Blood pressure, glycemia, albuminuria, weight, ASCVD risk	Treatment should be adapted to CKD stage and dialysis or transplant status
Frail or elderly CKD [83]	Individualized prevention	Functional status, nutrition, polypharmacy, life expectancy	Limited net benefit for primary prevention in some patients
Proteinuric or inflammatory phenotype [2,85]	Broader residual-risk assessment	Albuminuria, inflammation, endothelial dysfunction, cardiometabolic burden	Lipid therapy alone is insufficient
Severe HTG/chylomicronemia [79]	Pancreatitis-risk prevention	Early TG measurement, secondary causes, rapid TG reduction	Should be managed separately from routine ASCVD prevention

Abbreviations: ASCVD, atherosclerotic cardiovascular disease; CKD, chronic kidney disease; eGFR, estimated glomerular filtration rate; LDL-C, low-density lipoprotein cholesterol; mTOR, mammalian target of rapamycin; TG, triglycerides.

Implementation in routine practice is also shaped by biomarker availability, treatment burden, and access to newer therapies. ApoB is a validated measure of atherogenic particle number and can be useful when LDL-C is discordant with non-HDL-C or clinical risk, but routine use remains limited by reimbursement, clinician familiarity, and local laboratory reporting. For non-statin agents, practical barriers include cost, reimbursement rules, injection logistics, drug availability, administrative workload, and unequal access across healthcare systems, which may limit the use of PCSK9 inhibitors, inclisiran, bempedoic acid, or emerging triglyceride-lowering therapies even when they are clinically appropriate. In frail or elderly CKD patients, treatment escalation should also account for sarcopenia, falls, malnutrition, cognitive impairment, polypharmacy, adherence capacity, drug–drug interactions, and reduced medication clearance. Secondary ASCVD prevention generally remains a strong indication, but primary prevention requires a more selective approach, especially in dialysis-dependent, frail, or limited-life-expectancy patients. Medication review and, where appropriate, deprescribing should be considered when the expected benefit is unlikely to outweigh treatment burden, adverse effects, cost, or patient preference [83,85,86,87].

In diabetic kidney disease and proteinuric or inflammatory phenotypes, lipid care should be embedded within broader cardiometabolic and renal protection. Glycemic control, blood pressure management, albuminuria reduction, weight management, smoking cessation, and thrombotic-risk assessment influence residual risk alongside lipid therapy. Lipid indices such as non-HDL-C, ApoB, triglycerides, and remnant cholesterol may help identify higher-risk phenotypes, but they should not be treated as validated therapeutic targets for slowing renal decline. Severe HTG or chylomicronemia should be treated as a separate clinical priority because pancreatitis prevention may require more rapid triglyceride lowering and correction of secondary causes [70,79].

### 7.6. Circadian, Light-Exposure, and Physical-Activity Considerations

Circadian dysregulation, altered light exposure, and reduced physical activity may represent underrecognized contributors to cardiometabolic risk in CKD. The kidney has a strong peripheral circadian clock, and several renal functions, including electrolyte handling, urine concentration, blood pressure rhythmicity, erythropoietin biology, and phosphate metabolism, show time-dependent regulation. CKD is associated with disrupted sleep, altered activity rhythms, non-dipping blood pressure, and fragmentation of normal circadian patterns, while circadian disruption itself may worsen renal and cardiovascular physiology [88,89]. In advanced CKD and dialysis, these disturbances may be amplified by uremic symptoms, dialysis schedules, nocturia, daytime inactivity, reduced outdoor light exposure, inflammation, and comorbidity burden. Clinical dialysis data also show low daily activity, fewer steps on dialysis days, short sleep duration, and poor sleep quality in many HD patients, with early dialysis shifts appearing particularly disruptive for the sleep–wake cycle [90].

The mechanistic link with dyslipidemia remains incompletely defined, but several pathways are biologically plausible. Molecular clocks regulate metabolic homeostasis and interact with inflammation, sympathetic tone, feeding–fasting cycles, skeletal-muscle substrate use, and vascular function. In CKD, gut-derived uremic toxins may also interfere with circadian signaling through aryl hydrocarbon receptor activation, which can interact with brain and muscle ARNT-like protein 1 (BMAL1) and circadian locomotor output cycles kaput (Clock)/BMAL1 (activity and alter the amplitude or phase of circadian gene rhythms) [88,89]. This pathway is relevant to lipid metabolism because LPL activity, triglyceride-rich lipoprotein clearance, hepatic lipid synthesis, adipose tissue metabolism, and HDL remodeling are partly rhythm-dependent processes. Suppression or desynchronization of clock-gene signaling by uremic toxins, oxidative stress, and inflammation may therefore plausibly impair LPL rhythmicity and HDL function, although direct confirmation in CKD patients remains limited. This interpretation is consistent with kidney failure cohort data showing that several uremic toxins are inversely associated with conventional lipid biomarkers, including total cholesterol, non-HDL-C, LDL-C, and, in some analyses, HDL-C [91]. These mechanisms may indirectly affect triglyceride-rich lipoprotein handling, insulin resistance, HDL function, oxidative stress, and residual cardiovascular risk, although direct clinical evidence linking circadian disruption to specific lipid fractions in CKD remains limited. Therefore, circadian disruption should be interpreted as a potential modifier of metabolic risk rather than as a validated therapeutic target for dyslipidemia.

Physical activity is the most clinically actionable component of this axis. Patients with CKD are frequently inactive and may develop frailty, sarcopenia, reduced aerobic capacity, muscle weakness, and higher fall risk. Recent exercise guidance for CKD G3–G5 and G5D emphasizes that any activity is preferable to inactivity, that exercise prescriptions should be individualized, and that both home-based and intradialytic exercise can be integrated into routine care when clinically feasible. Exercise training improves aerobic capacity and physical function across CKD stages and dialysis populations, and some trials report favorable effects on inflammation, endothelial function, albuminuria, hospitalization, and quality of life, although effects on lipid parameters and non-traditional cardiovascular markers are not uniform [92]. Clinical exercise studies in dialysis populations have also reported improvements in lipid parameters, including reductions in triglycerides, total cholesterol, LDL-C, or lipid ratios and, in some cases, increased HDL-C, although study size, exercise modality, duration, and dialysis setting vary substantially. In this setting, regular physical activity, sleep regularity, daytime light exposure, and avoidance of prolonged sedentary time may be considered low-risk adjuncts to cardiometabolic care [92]. Timed physical activity, particularly when aligned with the patient’s dialysis schedule and daily energy pattern, may help reinforce rest–activity rhythms while also improving functional capacity. Daytime light exposure and more regular sleep timing may support circadian entrainment, but controlled CKD studies with lipid endpoints are still lacking. Vitamin D optimization may also be considered when deficiency is present, although it should be framed as correction of a common metabolic abnormality rather than as a specific lipid-lowering intervention [93].

These measures should therefore complement, not replace, guideline-directed lipid-lowering therapy and individualized ASCVD-risk management.

## 8. Knowledge Gaps and Future Directions

Several knowledge gaps remain in the interpretation and management of lipid disorders in renal failure. One major limitation is the incomplete representation of patients with CKD in cardiovascular randomized clinical trials. In a large contemporary assessment of cardiovascular trials, 74% excluded patients with CKD, and the proportion of trials excluding CKD increased over time. Separate results for patients with CKD were reported in only 13% of trials, while evidence was particularly scarce for eGFR < 30 mL/min/1.73 m^2^, dialysis recipients, and kidney transplant recipients [94]. This limits the direct applicability of lipid-lowering evidence to populations with the highest cardiovascular risk, where uremia, inflammation, vascular calcification, PEW, and competing non-atherosclerotic mechanisms may modify treatment response.

Another unresolved issue is the continued reliance on LDL-C as the dominant lipid marker. In renal failure, LDL-C may remain normal or even decrease, while atherogenic particle burden, TRLs, remnant particles, modified LDL, and dysfunctional HDL remain clinically relevant. Future risk models should evaluate non-HDL-C, ApoB, ApoB/ApoA-I ratio, Lp(a), oxidized and carbamylated LDL, and RC alongside conventional lipid fractions. Current pooled evidence indicates that higher RC is associated with greater odds of CKD, lower eGFR, and increased risk of progression to ESKD in T2DM-related CKD [49]. However, it remains uncertain whether RC is a causal mediator of renal injury, a marker of metabolic dysfunction, or both. Prospective studies and interventional trials are needed to determine whether reducing remnant particle burden can modify albuminuria, eGFR decline, renal fibrosis, or cardiovascular outcomes.

HDL biology also remains insufficiently translated into clinical practice. HDL-C concentration does not adequately reflect cholesterol efflux capacity, antioxidant activity, anti-inflammatory function, or lipid composition in CKD. Lipidomic approaches may help define dysfunctional HDL phenotypes, but current findings remain exploratory. In advanced CKD and kidney failure, targeted HDL lipid composition was not associated with endothelial dysfunction markers or unassisted arteriovenous fistula maturation, indicating either limited clinical utility of the tested lipidomic panel or the need for broader platforms, larger cohorts, and longitudinal designs [95].

Implementation gaps also need attention. ApoB, RC Lp(a), oxidized lipoproteins, and HDL function assays are not uniformly available, and CKD-specific thresholds for these markers remain poorly defined. Dialysis and transplant populations add further complexity because inflammation, nutrition, residual kidney function, dialysis modality, immunosuppressive therapy, and competing mortality risks can alter both lipid values and treatment response. Pragmatic trials, registry-based studies, and phenotype-stratified analyses could help clarify which biomarkers add actionable information beyond standard lipid panels.

Future research should move from isolated lipid targets toward integrated risk phenotyping. Lipid biomarkers should be studied together with inflammation, endothelial dysfunction, vascular calcification, albuminuria, dialysis modality, residual kidney function, nutritional status, frailty, and longitudinal renal trajectory. This approach may support a more precise model of lipid management in renal failure, in which treatment decisions reflect lipoprotein quantity, lipoprotein quality, uremic toxicity, residual inflammatory risk, and stage-specific CKD biology.

## 9. Conclusions

Lipid disorders in renal failure represent a complex, stage-dependent phenotype that cannot be interpreted through LDL-C values alone. Across the CKD continuum, impaired clearance of TRLs, remnant particle accumulation, small dense and modified LDL, HDL dysfunction, oxidative stress, inflammation, uremic toxicity, and endothelial injury interact with cardiovascular risk. These abnormalities may also contribute to renal lipotoxicity and CKD progression, although direct causal and therapeutic implications require further confirmation. Interpretation becomes more difficult in advanced CKD and dialysis, where malnutrition, PEW, inflammation, and non-atherosclerotic cardiovascular mechanisms may weaken the predictive value of conventional lipid markers.

Current evidence supports statin-based therapy, with or without ezetimibe, as the main pharmacological strategy for ASCVD risk reduction in most patients with non-dialysis CKD. By contrast, de novo statin initiation after dialysis onset remains less clearly supported. Non-statin therapies, including PCSK9-targeting agents, icosapent ethyl, bempedoic acid, and selected triglyceride-lowering strategies, may be useful in specific high-risk phenotypes, but evidence in advanced CKD, dialysis, and transplant populations remains limited.

Lipid management in renal failure should be individualized according to CKD stage, albuminuria, dialysis or transplant status, ASCVD burden, diabetes, frailty, nutritional status, inflammation, and treatment tolerance. Future work should refine integrated risk phenotyping by combining conventional lipid measures with ApoB, non-HDL-C, RC, Lp(a), HDL functionality, inflammatory markers, vascular calcification, and renal trajectory.

## Figures and Tables

**Table 1:** Typical lipid abnormalities across the CKD continuum.

CKD Stage/Clinical Setting	Typical Lipid Abnormalities	Main Contributing Factors	Clinical Interpretation
Early and moderate CKD	Mild-to-moderate HTG, reduced HDL-C, normal or mildly increased LDL-C, and early qualitative lipoprotein remodeling [23,24,25]. In diabetic kidney disease, elevated TG and reduced HDL-C are associated with albuminuria and eGFR decline [27].	Reduced LPL and HL activity, insulin resistance, inflammation, and impaired clearance of TRL [24,25,27].	Lipid abnormalities may appear before renal failure. TG, HDL-C, TG/HDL-C ratio, and particle remodeling may better reflect early cardiometabolic risk than LDL-C alone [24,25,26,27].
Advanced CKD and ESKD	Accumulation of TRLs and remnants, reduced HDL-C, altered HDL function, and normal or low LDL-C in some patients [10,28]. Low lipid values may coexist with inflammation, malnutrition, or PEW [29,30].	Uremia, oxidative stress, chronic inflammation, impaired LPL activity, reduced antioxidant defenses, malnutrition–inflammation states, and delayed clearance of TG-rich particles [10,28,29].	Conventional lipid markers become harder to interpret. Lower cholesterol may reflect inflammatory or malnutrition-prone states rather than reduced cardiovascular risk [30,31].
Hemodialysis	Frequent HTG, elevated VLDL-C, reduced HDL-C, impaired HDL function, and delayed clearance of triglyceride-rich particles [28].	Uremic toxin accumulation, oxidative stress, chronic inflammation, dialysis-related metabolic stress, impaired LPL activity, and reduced LCAT activity [28].	Lipid values should be interpreted together with inflammation, nutritional status, dialysis exposure, and cardiovascular risk, rather than through general-population LDL-C assumptions [28,30].
Peritoneal dialysis	Often more atherogenic than HD, with higher total cholesterol, TG, LDL-C, VLDL/remnant burden, and variable HDL-C findings across cohorts [32,33].	Continuous glucose absorption from PD solutions, insulin resistance, hepatic lipogenesis, peritoneal protein loss, residual kidney function, membrane characteristics, and treatment-related metabolic exposure [33,34].	PD-related dyslipidemia reflects systemic cardiovascular risk and modality-specific metabolic effects. Glucose exposure appears relevant, although lipid associations are not uniform across all fractions [33,34].
Kidney transplant recipients	Increased total cholesterol, LDL-C, VLDL-C, and TG, with variable HDL-C changes depending on patient profile, graft function, and therapy [36,37].	Corticosteroids, calcineurin inhibitors, mTOR inhibitors, post-transplant weight gain, adiposity, metabolic syndrome, post-transplant diabetes risk, residual graft dysfunction, and proteinuria [37,38,39,40].	Lipid interpretation should consider immunosuppressive regimen, weight trajectory, metabolic syndrome, graft function, proteinuria, and global cardiovascular risk. Dyslipidemia may affect both cardiovascular outcomes and graft prognosis [36,37,38,39,40].

Abbreviations: CKD, chronic kidney disease; eGFR, estimated glomerular filtration rate; ESKD, end-stage kidney disease; HDL-C, high-density lipoprotein cholesterol; LDL-C, low-density lipoprotein cholesterol; mTOR, mammalian target of rapamycin; PD, peritoneal dialysis; TG, triglycerides; VLDL-C, very-low-density lipoprotein cholesterol.

**Table 2:** Practical use of lipid biomarkers beyond LDL-C in CKD care.

Biomarker	How to Obtain It in Practice	When It Is Most Useful in CKD	Clinical Interpretation	Main Limitations
Non-HDL-C	Calculated from a standard lipid panel: total cholesterol − HDL-C.	Elevated triglycerides, diabetes, obesity, metabolic syndrome, albuminuria, or apparently acceptable LDL-C despite high ASCVD risk.	Reflects cholesterol carried by all atherogenic ApoB-containing particles, including LDL, VLDL, IDL, Lp(a), and remnants. It is often more informative than LDL-C alone when triglyceride-rich lipoproteins accumulate.	Does not directly measure particle number. Interpretation may be affected by inflammation, malnutrition, dialysis status, and non-fasting triglyceride variation.
ApoB	Requires a direct laboratory assay; often needs to be specifically requested.	Suspected discordance between LDL-C and atherogenic particle burden, especially in hypertriglyceridemia, diabetes, obesity, metabolic syndrome, nephrotic-range proteinuria, or advanced CKD.	Estimates the number of circulating atherogenic particles. Elevated ApoB despite acceptable LDL-C suggests residual particle burden and may support treatment intensification in high-risk patients.	Availability, reimbursement, assay use, and local reporting vary. CKD-specific treatment thresholds are not uniformly established.
Remnant cholesterol	Usually estimated rather than routinely reported; commonly calculated as total cholesterol − LDL-C − HDL-C when LDL-C is directly measured or reliably estimated.	Elevated triglycerides, diabetes, obesity, metabolic syndrome, CKD-related triglyceride-rich lipoprotein accumulation, or residual risk despite LDL-C control.	May identify residual remnant and triglyceride-rich lipoprotein burden. It should be interpreted as an adjunctive risk marker, not as a validated treatment target for CKD progression.	Calculation depends on lipid measurement method, fasting status, and LDL-C estimation accuracy. No lipid-lowering trial has yet shown that selective remnant cholesterol reduction improves renal outcomes as a primary endpoint.
Lp(a)	Direct assay, preferably once in adulthood; reporting in nmol/L is preferred when available.	Premature ASCVD, family history of ASCVD, progressive vascular disease, recurrent events despite LDL-C control, or unexpectedly high vascular risk.	Elevated Lp(a) supports more intensive management of modifiable ASCVD risk factors and may explain residual risk not captured by LDL-C.	Assays and units vary. Specific Lp(a)-lowering outcome therapies remain under investigation, and CKD-specific thresholds are not well established.
HDL function	Not routinely available; mainly research-based assessment of cholesterol efflux, antioxidant, and anti-inflammatory activity.	Research settings or mechanistic interpretation of CKD-related vascular risk, especially when HDL-C appears normal but inflammation is high.	Helps explain why HDL-C concentration may not reflect HDL quality in CKD. Dysfunctional HDL may lose protective vascular properties.	Not suitable for routine clinical decision-making. No standardized clinical assay or treatment threshold is available.

Note: This table reflects the authors’ practical interpretation of available evidence and guideline recommendations. Abbreviations: ApoB, apolipoprotein B; ASCVD, atherosclerotic cardiovascular disease; CKD, chronic kidney disease; HDL-C, high-density lipoprotein cholesterol; IDL, intermediate-density lipoprotein; LDL-C, low-density lipoprotein cholesterol; Lp(a), lipoprotein(a); VLDL, very-low-density lipoprotein.

**Table 3:** Major lipid-related mechanisms contributing to cardiovascular events in renal failure.

Mechanistic Domain	Lipid-Related Alteration in CKD/Renal Failure	Cardiovascular Implication	Main Cardiovascular Manifestations
ApoB-containing particle burden [4,12]	VLDL, IDL, LDL, remnants, and Lp(a) may remain clinically relevant even when LDL-C is normal or only mildly increased.	Increases arterial exposure to atherogenic particles and supports plaque initiation and progression.	CAD, ischemic heart disease, ASCVD events
Small dense and modified LDL phenotype [4,12,41]	CKD favors small dense LDL formation and increases susceptibility to oxidative and carbamylative modification.	Enhances endothelial entry, oxidative injury, foam-cell formation, and plaque vulnerability.	Coronary atherosclerosis, ACS, ASCVD
TRLs and remnants [4,12,49]	Impaired LPL activity, altered ApoC-III regulation, and delayed VLDL/remnant clearance promote accumulation of triglyceride-rich particles.	Sustains residual lipid risk through endothelial inflammation, oxidative stress, cytokine signaling, and remnant deposition.	CAD, atherosclerosis progression, residual cardiovascular risk
HDL dysfunction [4,12,46,50]	CKD impairs ApoA-I availability, LCAT activity, HDL maturation, cholesterol efflux, and antioxidant/anti-inflammatory HDL functions.	Weakens reverse cholesterol transport and may shift HDL toward a pro-inflammatory vascular phenotype.	Endothelial dysfunction, vascular inflammation, atherosclerosis progression
Lp(a) and oxidized phospholipid burden [12,51]	Lp(a) may accumulate with declining renal function and carries oxidized phospholipids linked to plaque biology.	Adds atherogenic, pro-inflammatory, and prothrombotic risk beyond conventional LDL-C measurement.	CAD, peripheral vascular disease, major adverse limb events
Uremic-inflammatory vascular injury [21,41,42]	Dyslipidemia interacts with uremic toxins, oxidative stress, chronic inflammation, endothelial dysfunction, and CKD-mineral and bone disorder.	Amplifies classical atherosclerosis and promotes impaired vasodilation, leukocyte adhesion, foam-cell formation, and procoagulant activation.	CAD, ACS, stroke, PAD, cardiovascular mortality
Vascular calcification and arterial stiffness [42,43,45]	Calcium–phosphate imbalance, oxidized lipids, loss of calcification inhibitors, and VSMC osteogenic transformation promote intimal and medial calcification.	Reduces vascular compliance, increases pulsatile load, and links plaque disease with calcific vasculopathy.	ACS, LVH, heart failure, valve calcification, cardiovascular mortality
Uremic cardiomyopathy and cardiac metabolic injury [42,44,45]	Uremic toxins, inflammation, oxidative stress, anemia, hypervolemia, insulin resistance, CKD-MBD, and calcification pathways contribute to myocardial remodeling.	Produces non-atherosclerotic cardiac injury that may coexist with lipid-driven atherosclerosis and vascular calcification.	Heart failure, arrhythmias, sudden cardiac death
Lipid paradox and protein-energy wasting in dialysis [30,46,47,48]	Low cholesterol or LDL-C in HD may reflect inflammation, malnutrition, dilutional hypolipidemia, sampling timing, or PEW rather than low vascular risk.	Limits direct extrapolation of general-population lipid thresholds to dialysis patients.	Cardiovascular mortality, all-cause mortality, ACS outcomes
Biomarkers beyond LDL-C [12,42,49,50,51]	Non-HDL-C, ApoB, ApoB/ApoA-I ratio, Lp(a), RC, oxidized phospholipids, and HDL functionality capture lipid risk not reflected by LDL-C alone.	Supports broader risk stratification when LDL-C is discordant with particle burden, inflammation, or dialysis status.	CAD, ACS, stroke, PAD, MACE

Abbreviations: ACS, acute coronary syndrome; ApoA-I, apolipoprotein A-I; ApoB, apolipoprotein B; ApoC-III, apolipoprotein C-III; ASCVD, atherosclerotic cardiovascular disease; CAD, coronary artery disease; CKD, chronic kidney disease; CKD-MBD, chronic kidney disease–mineral and bone disorder; HDL, high-density lipoprotein; IDL, intermediate-density lipoprotein; LCAT, lecithin–cholesterol acyltransferase; LDL, low-density lipoprotein; LDL-C, low-density lipoprotein cholesterol; Lp(a), lipoprotein(a); LPL, lipoprotein lipase; LVH, left ventricular hypertrophy; MACE, major adverse cardiovascular events; PAD, peripheral artery disease; PEW, protein-energy wasting; VLDL, very-low-density lipoprotein; VSMC, vascular smooth muscle cell.

**Table 4:** Pharmacological options for lipid management in patients with renal failure.

Therapy	Main Role in Renal Failure	Practical Clarification	Main Cautions/Limitations
Statins [6,70]	First-line ASCVD risk reduction in most patients with non-dialysis CKD.	Treatment is guided by global cardiovascular risk, CKD stage, diabetes, albuminuria, prior ischemic stroke, and established ASCVD rather than LDL-C alone.	Benefit is clearest before dialysis; statins are not primarily used to slow CKD progression.
Statin–ezetimibe therapy [6,70]	Additional LDL-C and non-HDL-C lowering in high-risk non-dialysis CKD.	Useful when lipid goals are not reached with statin alone or when high-dose statin escalation is undesirable.	Evidence is strongest in non-dialysis CKD; benefit after dialysis initiation is less certain.
PCSK9 monoclonal antibodies [73,74]	Additional LDL-C lowering in CKD patients with established ASCVD or very high ASCVD risk despite statin ± ezetimibe.	Evolocumab and alirocumab reduce LDL-C, total cholesterol, and Lp(a), with short-term renal stability reported across CKD stages, including stages 4–5.	Dialysis, transplant, long-term renal safety, and kidney-outcome data remain limited.
Inclisiran [75]	Emerging LDL-C-lowering option for ASCVD, hypercholesterolemia, or statin intolerance.	Twice-yearly maintenance dosing may support adherence in CKD patients with polypharmacy.	CKD-specific evidence remains limited; dialysis-specific and hard outcome data are sparse.
Bempedoic acid [76]	Oral LDL-C-lowering option for statin-intolerant high-risk patients.	May be useful when muscle-related statin intolerance limits therapy.	Small creatinine and blood urea nitrogen changes require cautious interpretation, especially in DKD or advanced CKD.
Conventional fibrates [10,78]	Treatment of clinically relevant HTG and TG-rich lipoprotein excess.	Best reserved for selected patients requiring TG lowering beyond LDL-C-directed ASCVD prevention.	Renal clearance, creatinine rise, eGFR reduction, dose adjustment, and myopathy risk limit use in advanced CKD, especially with statins.
Pemafibrate [10,75,78]	Selective PPARα modulator for TG lowering.	Mainly hepatically metabolized and may be considered when TG lowering is needed but renal tolerability is a concern.	CKD-specific cardiovascular outcome evidence remains limited; caution is still needed in severe renal dysfunction or macroalbuminuria.
Icosapent ethyl [80]	Residual cardiovascular-risk reduction in statin-treated patients with elevated TG and ASCVD or diabetes with additional risk factors.	Evidence supports use across a broad eGFR range, with larger absolute benefit in patients with eGFR < 60 mL/min/1.73 m^2^.	Monitor bleeding tendency and atrial fibrillation/flutter risk, especially at lower eGFR or with antithrombotic therapy.
Mixed EPA–DHA fish-oil supplementation [81]	Possible adjunct for inflammatory residual risk in HD.	May reduce CRP, although effects on IL-6 and TNF-α are inconsistent.	Formulations, doses, treatment duration, baseline inflammation, and comparators vary across trials.
Emerging ApoC-III /ANGPTL3-targeted therapies [10,79]	Potential future option for severe, persistent, or refractory HTG.	Most relevant for chylomicronemia phenotypes or recurrent pancreatitis risk.	Access, indication, genetic phenotype, and long-term CKD safety data remain limiting factors.

Abbreviations: ANGPTL3, angiopoietin-like 3; ApoC-III, apolipoprotein C-III; ASCVD, atherosclerotic cardiovascular disease; CKD, chronic kidney disease; CRP, C-reactive protein; DHA, docosahexaenoic acid; DKD, diabetic kidney disease; eGFR, estimated glomerular filtration rate; EPA, eicosapentaenoic acid; IL-6, interleukin-6; LDL-C, low-density lipoprotein cholesterol; Lp(a), lipoprotein(a); PCSK9, proprotein convertase subtilisin/kexin type 9; PPARα, peroxisome proliferator-activated receptor-α; TG, triglycerides; TNF-α, tumor necrosis factor-α.

## Data Availability

No new data were created or analyzed in this study.

## Abbreviations

The following abbreviations are used in this manuscript:

ACS	Acute coronary syndrome
ApoA-I	Apolipoprotein A-I
ApoB	Apolipoprotein B
ApoC-III	Apolipoprotein C-III
ASCVD	Atherosclerotic cardiovascular disease
CAD	Coronary artery disease
CKD	Chronic kidney disease
CVD	Cardiovascular disease
eGFR	Estimated glomerular filtration rate
ESKD	End-stage kidney disease
FAO	Fatty acid oxidation
GFR	Glomerular filtration rate
HD	Hemodialysis
HDL	High-density lipoprotein
HDL-C	High-density lipoprotein cholesterol
IDL	Intermediate-density lipoprotein
LCAT	Lecithin–cholesterol acyltransferase
LDL	Low-density lipoprotein
LDL-C	Low-density lipoprotein cholesterol
Lp(a)	Lipoprotein(a)
LPL	Lipoprotein lipase
MACE	Major adverse cardiovascular events
mTOR	Mammalian target of rapamycin
non-HDL-C	Non-high-density lipoprotein cholesterol
PAD	Peripheral artery disease
PCSK9	Proprotein convertase subtilisin/kexin type 9
PD	Peritoneal dialysis
PEW	Protein-energy wasting
RC	Remnant cholesterol
ROS	Reactive oxygen species
T2DM	Type 2 diabetes mellitus
TG	Triglycerides
TRL	Triglyceride-rich lipoprotein
VLDL	Very-low-density lipoprotein
VLDL-C	Very-low-density lipoprotein cholesterol
